# Clinical utility of genomic signatures in young breast cancer patients: a systematic review

**DOI:** 10.1038/s41523-020-00188-3

**Published:** 2020-09-25

**Authors:** Cynthia Villarreal-Garza, Ana S. Ferrigno, Cynthia De la Garza-Ramos, Regina Barragan-Carrillo, Matteo Lambertini, Hatem A. Azim

**Affiliations:** 1 Breast Cancer Center, Hospital Zambrano Hellion, Tecnologico de Monterrey, San Pedro Garza Garcia, Mexico; 2 Department of Oncology, Instituto Nacional de Ciencias Médicas y Nutrición Salvador Zubirán, Mexico City, Mexico; 3 Department of Medical Oncology, U.O.C. Clinica di Oncologia Medica, IRCCS Ospedale Policlinico San Martino, Genoa, Italy; 4 Department of Internal Medicine and Medical Specialties (DIMI), School of Medicine, University of Genova, Genoa, Italy

**Keywords:** Breast cancer, Cancer genomics

## Abstract

Risk stratification by genomic signatures has been shown to improve prognostication and guide treatment decisions among patients with hormone-sensitive breast cancer. However, their role in young women has not been fully elucidated. In this review, a systematic search was conducted for published articles and abstracts from major congresses that evaluated the use of genomic signatures in young breast cancer patients. A total of 71 studies were analyzed, including 561,188 patients of whom 27,748 (4.9%) were young. Women aged ≤40 years were subjected to genomic testing at a similar rate to older women but had a higher proportion of intermediate- to high-risk tumors when classified by EndoPredict (*p* = 0.04), MammaPrint (*p* < 0.01), and Oncotype DX (*p* < 0.01). In young women with low genomic risk, 6-year distant recurrence-free survival was 94%, while 5-year overall survival was nearly 100%. Nonetheless, young patients classified as low-risk had a higher tendency to receive chemotherapy compared to their older counterparts. In conclusion, genomic tests are useful tools for identifying young patients in whom chemotherapy omission is appropriate.

## Introduction

Young women with breast cancer (YWBC), defined as patients aged ≤40 years at diagnosis^1,2^, account for a variable proportion of patients diagnosed with breast cancer around the world. They comprise around 2–3% in developed regions such as the European Union and North America, but up to 12–14% in resource-constrained countries such as Latin America and Sub-Saharan Africa^3–5^. As a group, YWBC are characterized by an increased frequency of aggressive molecular subtypes^6–9^. This predisposes them to an increased risk of recurrence and shorter disease-free survival (DFS) compared to their older counterparts^9–12^.

The relatively worse prognosis of YWBC is particularly observed in hormone-receptor positive disease^9,11,13^. This has resulted in offering prolonged and intensive chemotherapy regimens to young patients, despite the lack of evidence that this improves their disease outcome^14^. The overtreatment of YWBC with chemotherapy can have a detrimental impact on their quality of life, as this group faces unique challenges related to chemotherapy-induced amenorrhea, infertility, and sexual dysfunction^15,16^. Thus, there is a need to refine the decision-making process to identify young patients who could safely forego adjuvant chemotherapy.

In the past decade, risk stratification by gene expression signatures has been endorsed by international guidelines to improve prognostication and guide treatment decisions among women with hormone-receptor positive breast cancer^17–19^. Most of these genomic signatures are commercially available, including: Oncotype DX, MammaPrint, EndoPredict, Prosigna, Breast Cancer Index (BCI), and Genomic Grade Index (GGI). However, these tests have been developed and validated in large cohorts mostly comprised of postmenopausal patients, hindering the extrapolation of solid conclusions about their performance in young women. We sought to address such limitations by performing a systematic review of studies that subjected YWBC to genomic testing.

## Results

**Fig. 1:**
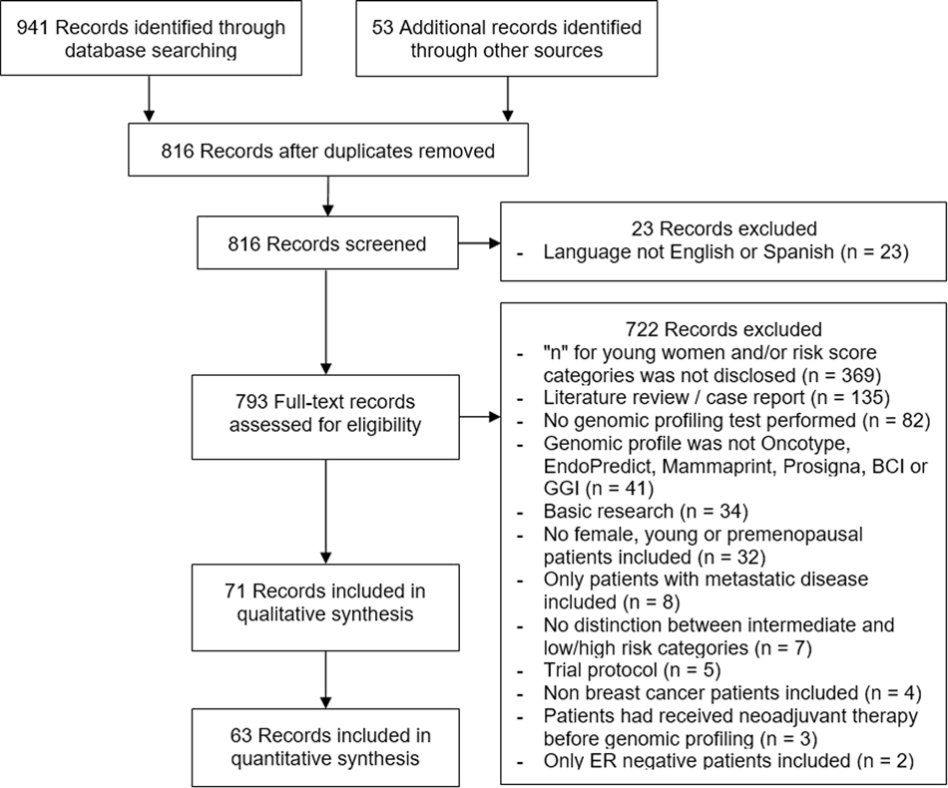
Preferred reporting items for systematic reviews and meta-analyses (PRISMA) flow diagram. Studies included in the qualitative but not in the quantitative synthesis are those that used data from the same sample of patients.

The search yielded a total of 861 original records, of which 71 studies were eligible and included in the analysis (Fig. 1). Most were cohort studies and had a quality of evidence level of I–II (72%). A list of the included studies is provided in Supplemental Table 1. These studies included a total of 561,188 patients who were subjected to genomic testing. In total, 540,647 patients were tested by Oncotype DX (96.3%), 18,614 by MammaPrint (3.3%), 1359 by EndoPredict (0.2%), 418 by GGI (0.1%), and 150 by BCI (0.03%). None of the studies that used Prosigna were eligible for this review.

### Representation of YWBC in genomic signature studies

The threshold used to define young age varied widely in the included studies. The age cut-off ranged from <35 to ≤55 years, with several defining “young” based on menopausal status (Table 1).

**Table 1:** Proportion of young patients participating in genomic risk trials stratified according to how “young age” was defined.

Definition of young used	Total # of participants	# of young patients (%)
<35 years	7,559	134 (1.8%)
≤35 years	471	22 (4.7%)
<40 years	444,070	14,946 (3.4%)
≤40 years	311,088	13,233 (4.3%)
<45 years	7,444	1,238 (16.6%)
<50 years	117,223	25,242 (21.5%)
≤50 years	13,111	4,431 (33.8%)
<55 years	142	67 (47.2%)
≤55 years	936	459 (49.0%)
Premenopausal	3,065	1,218 (39.7%)

Using the per-study definitions, 27,748 of the 561,188 evaluated patients (4.9%) were considered “young”. When considering exclusively those studies that defined “young” as aged ≤40 years at diagnosis, 13,233 of 311,088 patients (4.3%) fell into this category. Subgroup analyses in women ≤40 years were only available for Oncotype DX (*n* = 19,289, 5%), MammaPrint (*n* = 348, 2%), and EndoPredict (*n* = 34, 3%). None of the studies that utilized the other genomic tests considered in this review provided a dedicated analysis for YWBC.

### Influence of age on risk stratification by genomic tests

**Fig. 2:**
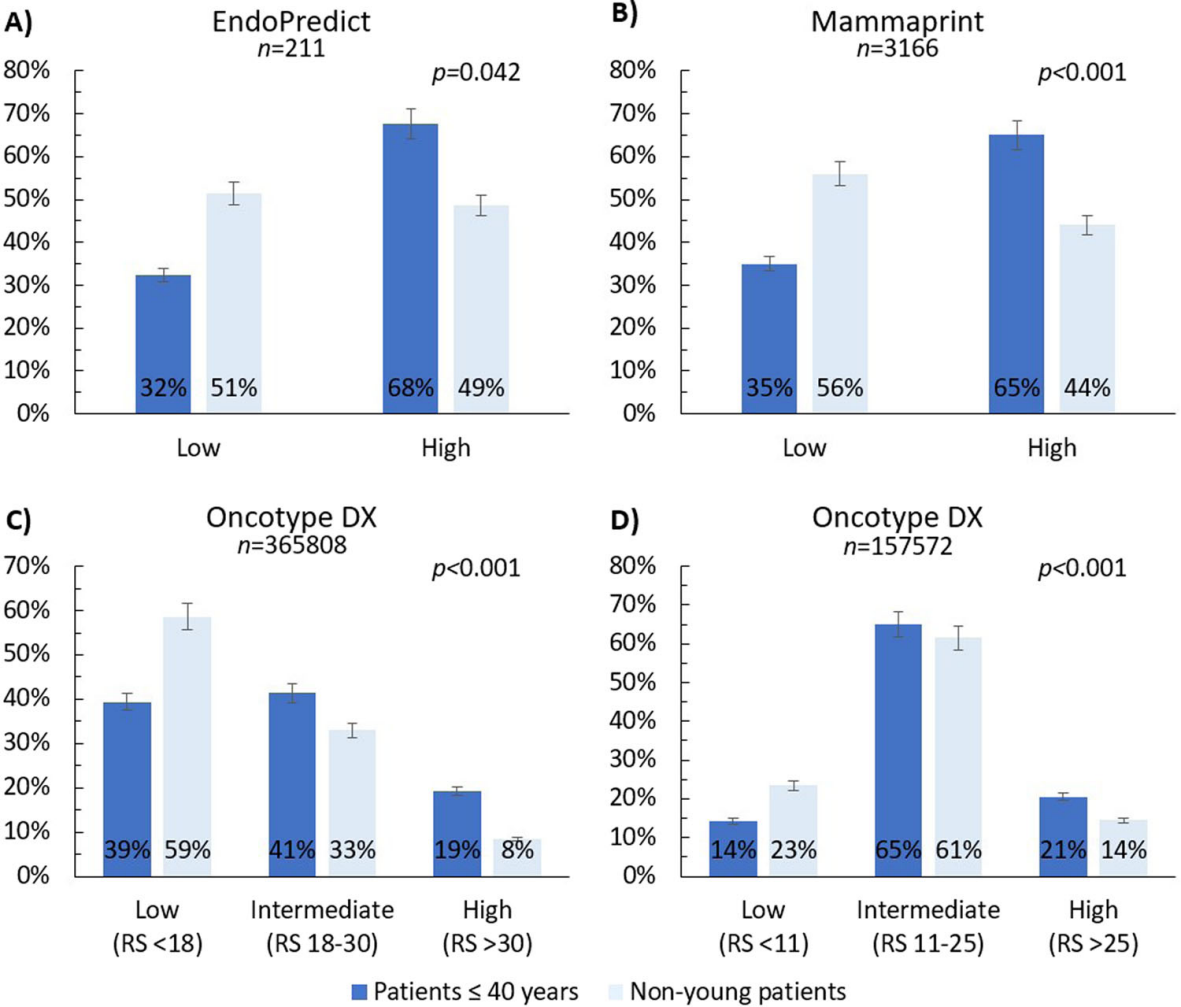
Proportion of patients in each genomic risk category according to age. **a** EndoPredict, **b** MammaPrint, **c** Oncotype DX (traditional recurrence risk categories), **d** Oncotype DX (TAILORx recurrence risk categories). Patients aged ≤40 years were predominantly classified as intermediate to high-risk by Oncotype DX (traditional cut-off value of >18:61%; TAILORx threshold of >11:86%) or as high-risk by MammaPrint (65%) and EndoPredict (68%). In contrast, patients aged >40 years were more likely to be assigned a low-risk category (59% when using the traditional threshold of Oncotype, 56% by MammaPrint, and 51% by EndoPredict). Error bars are set to 5%.

A larger proportion of high genomic risk tumors was observed in women ≤40 years compared to older groups across the three different genomic tests that provided a subgroup analysis: Oncotype DX (*p* < 0.001), MammaPrint (*p* < 0.001), and EndoPredict (*p* = 0.042) (Fig. 2). Notably, nearly two-thirds of tumors in patients ≤40 years were classified as high-risk by MammaPrint and EndoPredict, compared to around half in older patients.

### Impact of age on the decision to perform genomic tests

**Fig. 3:**
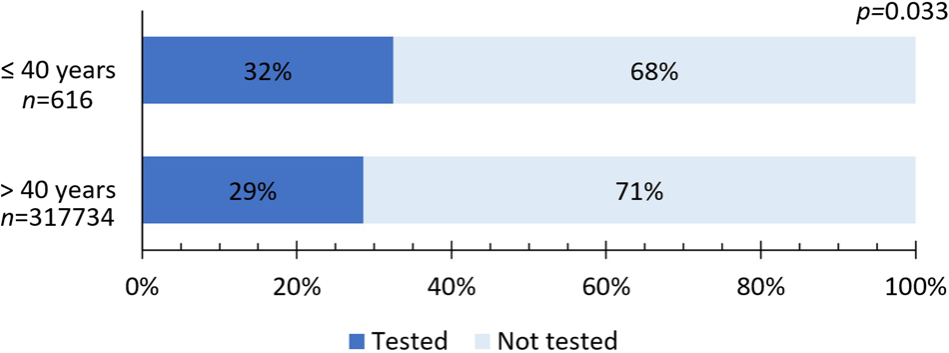
Proportion of patients that underwent genomic testing with Oncotype DX according to age. Data were extracted from studies by Poorvu et al.^21^, Lund et al.^20^, and Williams et al.^22^.

Only three studies compared the indication to perform genomic tests across age groups, all of which focused on the impact of age on Oncotype DX testing in the United States^20–22^. Overall, there was a tendency toward higher testing probability in younger patients (32 vs. 29%, *p* = 0.033) (Fig. 3).

### Prognostic value of genomic signatures in YWBC and potential impact of chemotherapy use

A total of nine studies evaluated the prognostic performance of genomic signatures in young women (using a per-study definition) and disclosed survival outcomes according to age group (Table 2). However, only two studies included in this review performed a dedicated analysis on the prognostic value of genomic signatures in women aged ≤40 years at diagnosis, both using Oncotype DX^21,23^.

**Table 2:** Studies investigating the impact of genomic test risk score and outcomes in young breast cancer patients^21,23,25,27,29,32,48–50^.

Trial	Genomic test (Oncotype DX recurrence risk categories, if applicable)	Definition of young used (*n*)	Outcome(s) measured	% risk of event for patients with low genomic risk (95% CI or ±SE)	% risk of event for patients with intermediate genomic risk (95% CI or ±SE)	% risk of event for patients with high genomic risk (95% CI or ±SE)
ECOG E2197: phase III trial^48^	Oncotype DX (<18, 18–30, >30)	<50 years (170)	Local recurrence at 10 years	1.9 (0.5–7.9)	8.1 (3.4–19.6)	8.3 (4.5–15.6)
			Loco-regional recurrence at 10 years	2.0 (0.5–7.9)	8.1 (3.4–19.6)	9.4 (5.3–16.5)
NSABP B-14 and B-28: phase III trials^49^	Oncotype DX (<18, 18–30, >30)	<50 years (339^a^)	Risk of distant recurrence after 5 years	NSABP B-28 5.3 (2.2–12.2) NSABP B-14 4.9 (1.6–14.4)	NSABP B-28 20.4 (12.6–32.1) NSABP B-14 25.2 (10.9–51.6)	NSABP B-28 23.3 (13.9–37.6) NSABP B-14 3.5 (0.5–23.1)
TAILORx: phase III trial^50^	Oncotype DX (<11, 11–25, >25)	≤50 years (3054)	Invasive disease-free survival at 9 years	Endocrine therapy arm 87.4 (±2.0)	Endocrine therapy arm Score 11–15: 85.7 (±2.2) Score 16–20: 80.6 (±2.5) Score 21–25: 79.2 (±3.3) Chemotherapy arm Score 11–15: 89.2 (±1.9) Score 16–20: 89.6 (±1.7) Score 21–25: 85.5 (±3.0)	Chemotherapy arm 80.3 (±2.9)
			Freedom from recurrence of breast cancer at a distant site at 9 years	Endocrine therapy arm 98.5 (±0.8)	Endocrine therapy arm Score 11–15: 97.2 (±1.0) Score 16–20: 93.6 (±1.4) Score 21–25: 86.9 (±2.9) Chemotherapy arm Score 11–15: 98.0 (±0.8) Score 16–20: 95.2 (±1.3) Score 21–25: 93.4 (±2.3)	Chemotherapy arm 88.7 (±2.1)
			Freedom from recurrence of breast cancer at a distant or local–regional site at 9 years	Endocrine therapy arm 95.4 (±1.3)	Endocrine therapy arm Score 11–15: 93.3 (±1.6) Score 16–20: 89.6 (±1.9) Score 21–25: 82.0 (±3.2) Chemotherapy arm Score 11–15: 94.4 (±1.5) Score 16–20: 93.0 (±)1.5 Score 21–25: 90.7 (±2.5)	Chemotherapy arm 86.1 (±2.2)
			OS at 9 years	Endocrine therapy arm 98.6 (0.9)	Endocrine therapy arm Score 11–15: 96.8 (±1.0) Score 16–20: 95.8 (±1.2) Score 21–25: 92.7 (±2.0) Chemotherapy arm Score 11–15: 97.5 (±0.9) Score 16–20: 96.1 (±1.2) Score 21–25: 93.9 (±1.9)	Chemotherapy arm 92.4 (±1.9)
TAILORx: phase III trial^25^	Oncotype DX (<11, 11–25, >25)	≤50 years (2958)	Recurrence, second primary cancer, or death at 9 years	Endocrine therapy arm: Low clinical risk 13.3 (±2.3) High clinical risk 9.3 (±4.5)	Endocrine therapy arm: Low clinical risk 17.4 (±1.8) High clinical risk 19.8 (±3.0) Chemotherapy arm: Low clinical risk 11.3 (±1.4) High clinical risk 13.5 (±3.0)	Chemotherapy arm: Low clinical risk 14.8 (±4.2) High clinical risk 24.0 (±4.2)
			Distant recurrence at 9 years	Endocrine therapy arm: Low clinical risk 1.8 (±0.9) High clinical risk 0 (±0)	Endocrine therapy arm: Low clinical risk 4.7 (±1.0) Clinical high-risk 12.3 (±2.4) Chemotherapy arm: Low clinical risk 3.9 (±1.0) High clinical risk 6.1 (±1.8)	Chemotherapy arm: Low clinical risk 6.2% (±2.5) High clinical risk 15.2% (±3.3)
HOHO: longitudinal cohort study^21^	Oncotype DX (<18, 18–30, >30)	≤40 years (577)	DRFS at 6 years	Lymph node-negative 97.5 (90.1–99.4) Lymph node-positive 85.9 (72.6–93.0)	Lymph node-negative 93.1 (86.0–96.7) Lymph node-positive 87.3 (76.0–93.5)	Lymph node-negative 86.4 (72.0–93.7) Lymph node-positive 62.8 (45.1–76.2)
HOHO: longitudinal cohort study^21^	Oncotype DX (<11, 11–25, >25)	≤40 years (577)	DRFS at 6 years	Lymph node-negative 94.4 (66.6–99.2) Lymph node-positive 92.3 (56.6–98.9)	Lymph node-negative 96.9 (92.7–98.7) Lymph node-positive 85.2 (75.3–91.4)	Lymph node-negative 85.1 (72.9–92.1) Lymph node-positive 71.3 (57.3–81.5)
Petkov et al.^32^	Oncotype DX (<11, 11–25, >25)	≤50 years (2588)	Breast cancer specific mortality at 5 years	No/unknown chemotherapy arm (±0)	No/unknown chemotherapy arm Score 11–15: 0.5 (±0.3) Score 16–20: 1.3 (±0.9) Score 21–25: 1.6 (±1.6) Chemotherapy arm Score 11–15: 2.3 (±1.4) Score 16–20: 1.6 (±0.9) Score 21–25: 1.2 (±1.2)	No/unknown chemotherapy arm 4.4 (±4.3) Chemotherapy arm 6.1 (±2.0)
Sammons et al.^23^	Oncotype DX (<11, 11–25, >25)	≤40 years (5899)	OS at 5 years	Recurrence score 0–25: 99 (ND)		Score: 26–30: 94 (ND) Score: 31–100: 92 (ND)
EORCT 10041/BIG 03-04 MINDACT: phase III trial^27^	MammaPrint	<45 years (1100)	DMFS at 5 years	Low clinical risk 98.3 (96–99.3) High clinical risk 97.4 (90–99.4)	N/A	Low clinical risk 95.5 (91.6–97.7) High clinical risk 89.2 (85.6–92.0)
GEICAM 9906: phase III trial^29^	EndoPredict	Premenopausal (300)	DMFS at 10 years	93 (ND)	N/A	67 (ND)

*CI* confidence interval, *SE* standard error, *N/A* not applicable, *ND* not disclosed, *DMFS* distant metastasis-free survival, *DRFS* distant recurrence-free survival, *OS* overall survival.

Only those with ^a^high ESR1 expression were taken into account.

### Oncotype DX

Using the TAILORx thresholds (i.e., <11, 11–25, and >25), Poorvu et al.^21^ evaluated the prognostic performance of Oncotype DX in breast cancer patients aged ≤40 years at diagnosis. Six-year distant recurrence-free survival (DRFS) for patients with N0 disease were 94.4% for low, 96.9% for intermediate, and 85.1% for high genomic risk tumors (*p* = <0.001). The proportion of patients that received chemotherapy for each risk category was 21.2%, 44.1%, and 91.7%, respectively. Remarkably, patients with N0 disease with low to intermediate genomic risk demonstrated excellent outcomes. Particularly, chemotherapy use was not associated with better DRFS in the intermediate group (*p* = 0.25). On the other hand, for patients with N1 disease, most of whom were treated with chemotherapy, 6-year DRFS rates were 92.3, 85.2, and 71.3% for each risk category. In a multivariate analysis that included tumor size, node status, histological grade and chemotherapy use, a high genomic risk score was found to be associated with the risk of distant recurrence (hazard rate_recurrence score ≤25 vs. >25_ 0.31; *p* = 0.01).

Sammons et al.^23^ analyzed data from patients with stage I–II, hormone-receptor positive/HER2-negative, N0 disease with documented Oncotype DX score in the National Cancer Database. They found that women aged ≤40 years with a low to intermediate score using TAILORx thresholds (i.e., ≤25) had an excellent 5-year overall survival (OS) despite low chemotherapy use, with no differences according to risk category (99%; *p* = 0.93). In patients with a high genomic risk for recurrence (i.e., >25), the 5-year OS was significantly lower (94% for those with a recurrence score of 26–30 and 92% for >30) even though the majority received chemotherapy, with an estimated hazard ratio_high vs. low risk_ of 5.13 (*p* < 0.001).

Other noteworthy analyses of prognostic value of Oncotype DX in YWBC include Harbeck et al.^24^ who demonstrated that patients aged <40 years with a high recurrence score using the TAILORx threshold (i.e., >25) had a similar DFS than their older counterparts when treated with chemotherapy, and Sparano et al.^25^ who showed that patients aged ≤40 years who had high-intermediate risk scores (i.e., 16–25) did not benefit from chemotherapy addition in terms of DFS.

### MammaPrint

Of the articles included in this review, none examined the prognostic value of MammaPrint in women aged ≤40 years. However, its prognostic performance in young patients (using a per-study definition) was evaluated in the MINDACT phase III trial, which assessed the clinical utility of genomic signatures when recommending adjuvant chemotherapy for patients with stage T1–2 or operable T3 disease^26^. This study included 2226 (33%) patients aged <50 years, with only 122 (1.8%) aged <35. The participants were distributed into four groups: clinical-low and genomic-low (CL/GL), clinical-low and genomic-high (CL/GH), clinical-high and genomic-low (CH/GL), and clinical-high and genomic-high (CH/GH) risk. All women in the CL/GL group did not receive chemotherapy, while those in the CH/GH group did. Patients with discordant risk results were randomized to either receive or abstain from chemotherapy.

In a post hoc analysis, Aalders et al.^27^ found that the use of MammaPrint reduced the proportion of patients aged <45 years classified as high-risk compared to relying only on clinical parameters (61% CH vs. 48% GH). In addition, MammaPrint added important prognostic information in women aged <45 years, particularly in the CH group, as sub-classification by genomic score translated into a 5-year distant metastasis-free survival (DMFS) of 95.5% for the CH/GL, compared to 89.2% in the CH/GH category. For patients in the CL group, prognosis was good irrespective of MammaPrint results with DMFS rate of 98.3% and 97.4% for the GL and GH groups, respectively.

In a subsequent analysis, Piccart et al.^28^ reported that in patients aged 50 years or younger within the CH/GL group, treatment with endocrine therapy alone demonstrated a non-statistically significant trend toward worse outcomes compared to chemotherapy (DMFS absolute difference of 3% at 5 years in women aged ≤50 years vs. 0.2% in older patients).

### EndoPredict

None of the studies included in this review examined the prognostic value of EndoPredict in women aged ≤40 years. Nonetheless, the prognostic value of EndoPredict in premenopausal women was evaluated by Martin et al.^29^ in the GEICAM 9906 trial, which is a phase III clinical trial that compared two adjuvant chemotherapy regimens in patients with hormone-receptor-positive/HER2-negative, lymph node-positive disease. Of the 555 patients that underwent genomic testing, 300 (54%) were premenopausal. DMFS at 10 years in this subgroup was found to be 93% for those with low-risk vs. 67% for high-risk scores (*p* < 0.0001). The prognostic information provided by EndoPredict was determined to be independent of age (<50 vs. ≥50 years), tumor grade, lymph node status, tumor size, hormone-receptor expression, and Ki67.

### Chemotherapy use according to genomic risk stratification and age

**Fig. 4:**
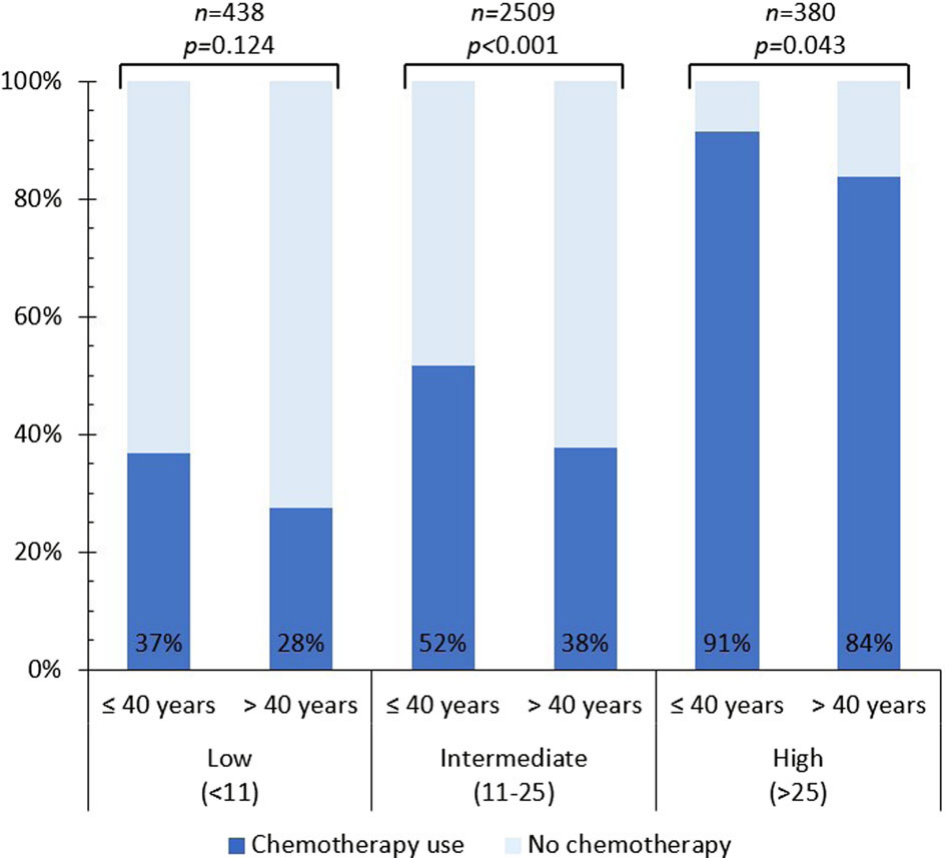
Proportion of patients that received chemotherapy according to Oncotype DX recurrence score and age. Data were extracted from studies by and Barcenas et al.^33^, Petkov et al.^32^, and Poorvu et al.^31^.

Despite the available evidence of the prognostic value of genomic risk stratification in young patients, few studies have explored the impact of the risk categories on the use of chemotherapy in this group. Five studies explored chemotherapy use according to genomic risk stratification by Oncotype DX in women aged ≤40 years at diagnosis, of which only three disclosed the number of women stratified to each risk category (Fig. 4). In addition, one study explored chemotherapy use among patients with a low risk for recurrence according to EndoPredict.

Namuche et al.^30^ included 53 YWBC and 498 older patients with early stage breast cancer in a multicenter retrospective study and found that those in the low-risk category received more chemotherapy than older patients (28 vs. 11.3%, *p* = 0.037). Nonetheless, chemotherapy use was similar in both age groups for patients in the intermediate-risk category (*p* = 0.484).

Poorvu et al.^31^ analyzed data from 182 YWBC in their prospective cohort that had Oncotype DX performed as part of their clinical care. Using traditional thresholds for recurrence risk (i.e., <18, 18–30, and >30), 24% of patients with a low, 57% with an intermediate, and 100% with a high recurrence score received chemotherapy. With TAILORx thresholds (i.e., <11, 11–25, and >25), the proportion of patients that received chemotherapy were 24%, 39%, and 87%, respectively.

As previously mentioned, Sammons et al.^23^ analyzed treatment strategy according to Oncotype DX risk stratification in women aged ≤40 years with N0 disease in the National Cancer Database and found that the proportion of patients that received chemotherapy was 6.4% of those with a recurrence score between 0 and 10, 11.4% between 11 and 15, 31.7% between 16 and 20, 59.9% between 21 and 25, 79.8% between 26 and 30, and 88.7% >30. Similarly, Petkov et al.^32^ analyzed data from young women with hormone-receptor-positive/HER2-negative, lymph node-positive disease who had undergone Oncotype DX and were documented in the Surveillance, Epidemiology, and End Results Program database. They found that age ≤40 years, large tumor size and high histological grade are associated with increased use of chemotherapy. Noteworthy, approximately 43% of YWBC with a low-risk score (i.e., <11) received chemotherapy compared with 28% of the older patients in this risk category (*p* = 0.03).

Lastly, Barcenas et al.^33^ conducted a single center retrospective analysis on patients with stage I–II, hormone-receptor-positive/HER2-negative, N0 disease who had an Oncotype DX test performed. Among patients with an intermediate score according to TAILORx thresholds (i.e., 11–25), it was found that patients who were treated with chemotherapy were younger (38% of patients aged ≤40 years received chemotherapy vs. 15% of their older counterparts), had larger tumors and a higher clinical stage.

On the other hand, Villarreal-Garza et al.^34^ examined the use of chemotherapy according to risk stratification by EndoPredict and found that 1/11 (9%) of women aged ≤40 years and 4/35 (11%) of older premenopausal patients were recommended chemotherapy by the institutional tumor board despite having a low EPclin risk score.

## Discussion

YWBC constituted ~4% of patients included in studies evaluating the role of genomic tests in breast cancer. This is comparable to the prevalence of YWBC in developed nations^35,36^. While this suggests that YWBC might not be under-represented in clinical studies, such low prevalence hinders the individual trials to perform statistically reliable subgroup analyses of these patients. Noteworthy, only 5% of the evaluated studies had a main objective focused on young women^22,37,38^. In the current systematic review, we have tried to address such limitations by performing a pooled analysis to refine the knowledge regarding the role of genomic tests in YWBC.

The results showed that young women are more commonly diagnosed with high genomic risk tumors compared to their older counterparts. This is consistent with previous studies showing a higher prevalence of luminal-B tumors in YWBC^6,8,9,39^. Of note, it has been previously shown that hormone-receptor-positive tumors arising in young women are more commonly enriched with a GATA3 mutation^40^, which has been proposed to predispose to endocrine resistance^41,42^. YWBC also present a lower prevalence of PIK3CA mutations^40,42^, which have been associated with better prognosis^43^. Taken together, it is conceivable that hormone-receptor-positive tumors arising in YWBC are predominantly classified as high-risk, reflecting the aggressive biological behavior of these tumors.

Recently, the TAILORx and MINDACT phase III trials established the role of Oncotype DX and MammaPrint as reliable tools to determine the need for adjuvant chemotherapy^26,44^. However, their subgroup analyses in premenopausal patients have steered major controversy. In the TAILORx trial, it was shown that women ≤50 years with a high-intermediate recurrence score (i.e., 21–25) and those with a recurrence score between 16 and 20 with a high clinical risk benefit from adjuvant chemotherapy^25^. This subgroup analysis was not in line with the main analysis, which showed endocrine therapy alone was as good as chemotherapy in intermediate-risk patients^44^. Furthermore, the benefit of chemotherapy in the high-intermediate risk category (i.e., 16–25) was only observed in patients aged 41–45 years and premenopausal patients between 46 and 50 years but not in the group aged ≤40 years^25^. This observation is hard to reconcile especially in light of the trial by Poorvu et al.^21^ who did not observe a benefit from chemotherapy use in terms of DRFS at 6 years for YWBC with an intermediate risk for recurrence (i.e., 11–25). On the other hand, in the MINDACT trial patients aged ≤50 years with CH/GL risk appeared to derive more benefit of chemotherapy^28,45^, which was also different to the main results taking into account data from all patients^26^.

To put this data into context, several points need to be considered. First, it is worth noting that in both the TAILORx and MINDACT trials, subgroup analysis according to age were not preplanned and, in some cases, did not reach statistical significance, making it hard to justify challenging the clinical utility of these genomic tests in younger women based solely on these analyses. Second, the recent data from SOFT and TEXT trials established ovarian function suppression (OFS) in combination with either tamoxifen or exemestane as superior treatment options to tamoxifen alone^46,47^. Thus, it remains questionable if the majority of YWBC randomized to endocrine therapy alone in these trials were adequately treated. Of note, only 15% and 8% of patients treated with OFS, in TAILORx and MINDACT trials, respectively.

Accordingly, it is reasonable to deduct that it is unlikely that chemotherapy offers a clinically relevant difference in survival over adequate endocrine therapy alone in patients with an intermediate genomic recurrence risk score. Nevertheless, it is relevant to share this uncertainty with YWBC taking into account various quality of life considerations, which vary from one patient to the other. In addition, the integration of other clinicopathological risk factors would possibly be needed in order to reach an adequate tailored decision for each young patient.

Notably, we found there is a trend for a higher proportion of YWBC to receive adjuvant chemotherapy compared to their older counterparts, even when classified as low genomic risk. This underscores a general perception that young age per se is an indication for more aggressive treatment, a notion that has been strongly challenged by several consensus groups^1,17^. Recently, in a dedicated prospective study by Poorvu et al.^21^, chemotherapy use was not associated with improved outcomes in patients classified as intermediate recurrence risk by Oncotype DX. Furthermore, in the SOFT and TEXT trials, the 5-year DFS of premenopausal patients treated with endocrine therapy alone was close to 95%^46^. These patients were mostly classified as low-risk by clinical parameters and comprised 43% of the cohort. This highlights that adequate endocrine therapy alone could achieve excellent outcomes, provided that eligible patients are well identified.

### Limitations

There are several limitations of this review. First, the search was designed to identify only those articles that included determined words in the title and keywords sections, hence articles that did examine the performance of genomic tests in patients with breast cancer but did not meet the search criteria could have been inadvertently missed. Efforts to attenuate the risk of missing important information were made by cross-referencing and searching the proceedings of relevant annual meetings, but this was a non-systematic measure. Second, eligible studies were only those that disclosed the genomic risk distribution of young participants. Thus, studies that included young patients but did not disclose this information were excluded from the analysis. Third, the study designs, inclusion criteria, and definition of YWBC of the studies in this review varied; this could have treatment and prognostic implications that limit the ability of drawing firm conclusions when synthetizing data. Fourth, even though the objective of this study was to analyze the utility of commercial genomic assays in the management of YWBC, most of the information available corresponded to Oncotype DX. Lastly, this analysis was performed on published data and the possibility that data from the same patient was included in more than study cannot be excluded. To control for this potential source of bias, studies by the same group were cross-checked and data with significant overlap was excluded from the quantitative analyses.

## Conclusions

In conclusion, current data support that genomic signatures provide comparable prognostic information in YWBC compared to older counterparts and remain an important tool to refine the decision-making process. However, it appears that the medical community is reluctant to rely upon genomic risk stratification to forego chemotherapy in YWBC given the inherent poor prognosis observed in this subgroup. Available evidence challenges this notion. Considering the unique quality of life issues related to managing YWBC, the Breast Cancer in Young Women Consensus endorses the discussion of chemotherapy omission in cases with a low-risk genomic profile^17^. In the intermediate-risk group, a “one-size fits all” approach should not be used, instead several considerations should be taken into account to individualize the treatment decision.

## Methods

This is a systematic review aiming to evaluate:

1. The representation of YWBC in clinical studies assessing the role of genomic tests.
2. The genomic risk stratification of YWBC compared to their older counterparts.
3. The impact of age on performing genomic tests in routine clinical practice.
4. The prognostic performance of genomic tests in YWBC.
5. The impact of age on the use of adjuvant chemotherapy in the era of genomic tests.

A literature search was conducted in the MEDLINE, EMBASE, and CENTRAL databases from their inception up to October 3, 2019 using the following keywords: “breast cancer” or its synonyms “breast carcinoma”, “breast neoplasm”, “breast malignancy”, or “breast tumors” and the denomination of “genomic signature” or its equivalents “21-gene”, “70-gene”, “multigene”, “Oncotype DX”, “Oncotype”, “EndoPredict”, “MammaPrint”, “Prosigna”, “PAM50”, “breast cancer index”, “BCI”, “genomic grade index”, or “GGI” in the title section. The search was not limited by date of publication, type of study, or language. Cross-referencing was performed to retrieve relevant articles that might have been missed. In addition, a search was performed in the proceedings of the 2016–2019 American Society of Clinical Oncology, European Society for Medical Oncology, and San Antonio Breast Cancer Symposium annual meetings to retrieve abstracts that met the selection criteria.

Eligible studies were those that presented original findings, were published in English or Spanish, performed any of the genomic tests listed above, included YWBC, and disclosed the number of patients per risk category. Potentially eligible articles were evaluated independently by two authors (A.S.F. and C.D.G.R.) and defined variables were extracted in duplicate into an electronic database developed specifically for this review. The extracted variables included study design, name of the genomic test, inclusion criteria, definitions used to determine genomic risk categories, total number of participants, definition used for YWBC, number of participating young women, distribution of patients across genomic risk categories, outcome measured, and median follow-up. In addition, the quality of evidence was evaluated using the Oxford Centre for Evidence-Based Medicine 2009 criteria. Disagreements were resolved by a third author (CVG). No assessment for risk of bias was performed.

Statistical analysis was carried out using the SPSS Statistics software (IBM Corp., Armonk, N.Y., USA) and Pearson’s *χ*^2^ tests were applied to explore differences in the distribution of categorical variables. When information was available, the analysis was focused on YWBC using the definition of women aged ≤40 years at diagnosis. Statistical significance was defined as *p* < 0.05.

## Supplementary information

Supplemental Table 1

## Data Availability

All data generated or analyzed during this study are available upon reasonable request to the corresponding author.
