# Supplementary material for: Clinical utility of genomic signatures in young breast cancer patients: a systematic review
Source: NPJ Breast Cancer. 2020 Sep 25;6:46. doi: 10.1038/s41523-020-00188-3 (PMC7519162; doi:10.1038/s41523-020-00188-3)
Supplement: Supplementary file 1 — Supplemental Table 1 [file 41523_2020_188_MOESM1_ESM.pdf]

Supplementary Table 1. Studies included in the systematic review.

Reference	Genomic test	Total number of participants	Age threshold used to define young	n (%) of young participants	Study design (level of evidence)
Curtit E, Vannetzel JM, Darmon JC, et al. Results of PONDx, a prospective multicenter study of the Oncotype DX((R)) breast cancer assay: Real-life utilization and decision impact in French clinical practice. Breast. 2019;44:39-45.	Oncotype*	866	< 35 years	12 (1%)	Prospective - multicenter (1)
Zeng Y, Li Q, Qin T, et al. Impact of a 21-Gene Recurrence Score Test on the Choice of Adjuvant Chemotherapy for Hormone Receptor-positive Early-stage Breast Cancer: A Prospective Study. Anticancer Res. 2017;37(8):4539-4547.	Oncotype*	227	≤ 35 years	16 (15%)	Prospective – single center (1)
Gwin K, Pinto M, Tavassoli FA. Complementary value of the Ki-67 proliferation index to the oncotype DX recurrence score. Int J Surg Pathol. 2009;17(4):303-310.	Oncotype*	32	< 40 years	1 (3%)	Retrospective – single center (4)
Harbeck N, Gluz O, Wuerstlein R, et al. Abstract P1-06-06: No age-related outcome disparities according to 21-gene recurrence score groups in early breast cancer patients treated by adjuvant chemotherapy in the prospective WSG PlanB trial. Cancer Research. 2018;78(4 Supplement):P1-06-06-P01-06-06.	Oncotype†	2,577	< 40 years	111 (4%)	Prospective – single center (1)
Ibraheem AF, Press DJ, Olopade OI, Huo D. Community clinical practice patterns and mortality in patients with intermediate oncotype DX recurrence scores: Who benefits from chemotherapy? Cancer. 2019;125(2):213-222.	Oncotype	73,185	< 40 years	2,493 (3%)	Retrospective – registry-based (2)
Lund MJ, Mosunjac M, Davis KM, et al. 21-Gene recurrence scores: racial differences in testing, scores, treatment, and outcome. Cancer. 2012;118(3):788-796.	Oncotype*	272	< 40 years	11 (4%)	Retrospective – multicenter (2)
Paik S, Shak S, Tang G, et al. A multigene assay to predict recurrence of tamoxifen-treated, node-negative breast cancer. N Engl J Med. 2004;351(27):2817-2826.	Oncotype*	668	< 40 years	59 (9%)	Prospective – multicenter (1)

Stemmer SM, Klang SH, Ben-Baruch N, et al. The impact of the 21-gene Recurrence Score assay on clinical decision-making in node-positive (up to 3 positive nodes) estrogen receptor-positive breast cancer patients. Breast Cancer Res Treat. 2013;140(1):83-92.	Oncotype*	282	< 40 years	6 (2%)	Retrospective – multicenter (2)
Swain SM, Nunes R, Yoshizawa C, Rothney M, Sing AP. Quantitative Gene Expression by Recurrence Score in ER-Positive Breast Cancer, by Age. Adv Ther. 2015;32(12):1222-1236.	Oncotype*	362,001	< 40 years	11,983 (3%)	Cross-sectional (4)
Turashvili G, Chou JF, Brogi E, et al. 21-Gene recurrence score and locoregional recurrence in lymph node-negative, estrogen receptor-positive breast cancer. *Breast Cancer Res Treat.* 2017;166(1):69-76.	Oncotype*	2,326	< 40 years	128 (6%)	Retrospective – single center (2)
Zimmerman BS, Cascetta KP, Ru M, et al. Abstract P1-07-16: Retrospective analysis of oncotype DX recurrence score (RS) and discordance in patients with node-negative, ER+ breast cancer with recurrence. Cancer research. 2018;78(4 Supplement):P1-16.	Oncotype	13	< 40 years	1 (8%)	Retrospective – single center (3)
Barcenas CH, Raghavendra A, Sinha AK, et al. Outcomes in patients with early-stage breast cancer who underwent a 21-gene expression assay. Cancer. 2017;123(13):2422-2431.	Oncotype†	1,424	≤ 40 years	105 (7%)	Retrospective – single center (2)
Krystel-Whittemore M, Brogi E, Bowser ZL, Dickler M, Hudis C, Wen HY. Distant metastases in breast cancer patients with Oncotype Dx recurrence score lower than 18. Modern Pathology. 2016;29:28–80.	Oncotype	7	≤ 40 years	4 (57%)	Retrospective – single center (3)
Sammons S, Y R, J F, et al. Characterization of Oncotype DX recurrence score (RS) and chemotherapy utilization patterns in young women (≤40) with early stage ER+/HER2-, lymph node negative breast cancer. 2019 San Antonio Breast Cancer Symposium; 2019; San Antonio, TX, USA.	Oncotype†	150,023	≤40 years	5,899 (4%)	Retrospective – registry-based (2)
Sparano JA, Gray RJ, Makower DF, et al. Prospective Validation of a 21-Gene Expression Assay in Breast Cancer. N Engl J Med. 2015;373(21):2005-2014.	Oncotype†	10,253	≤ 40 years	377 (4%)	Prospective – multicenter (1)
Toole MJ, Kidwell KM, Van Poznak C. Oncotype dx results in multiple primary breast cancers. Breast Cancer (Auckl). 2014;8:1-6.	Oncotype	45	≤ 40 years	3 (7%)	Retrospective – single center (4)

Petkov VI, Kurian AW, Jakubowski DM, Shak S. Breast Cancer-specific Survival in Patients Age 50 Years or Younger With Node-positive Breast Cancer Treated Based on the 21-Gene Assay in Clinical Practice. 2019 San Antonio Breast Cancer Symposium; 2019; San Antonio, TX, USA.	Oncotype†	2,588	≤40 years	436 (17%)	Retrospective – registry-based (2)
Poorvu PD, Gelber SI, Rosenberg SM, et al. Prognostic impact of the 21-gene recurrence score assay among young women with node-negative and node-positive ER+/HER2- breast cancer. 2018 San Antonio Breast Cancer Symposium; 2018; San Antonio, TX, USA.	Oncotype*,†	509	≤ 40 years	509 (100%)	Prospective – multicenter (2)
Poorvu PD, Gelber SI, Rosenberg SM, et al. Prognostic Impact of the 21-Gene Recurrence Score Assay Among Young Women With Node-Negative and Node-Positive ER-Positive/HER2-Negative Breast Cancer. J Clin Oncol. 2019:JCO1901959.	Oncotype*,†	509	≤40 years	509 (100%)	Prospective – multicenter (2)
Poorvu PD, Gelber SI, Rosenberg SM, et al. Selection for Oncotype Dx testing among young women with early-stage ER+/HER2- breast cancer. Journal of Clinical Oncology. 2018;36(15_suppl):533-533.	Oncotype*,†	182	≤ 40 years	182 (100%)	Prospective – multicenter (1)
Shak S, Baehner F, Stein M, et al. Abstract P3-10-01: Quantitative Gene Expression Analysis in a Large Cohort of Estrogen-Receptor Positive Breast Cancers: Characterization of the Tumor Profiles in Younger Patients (≥40 yrs) and in Older Patients (≥70 yrs). Cancer Research. 2010;70(24 Supplement):P3-10-01-P13-10-01.	Oncotype*	145,240	≤ 40 years	5,794 (4%)	Cross-sectional (4)
Tozbikian GH, Zynger DL. HER2 equivocal breast cancer that is positive by alternative probe HER2 FISH are classified as HER2 negative by Oncotype DX. Breast J. 2018;24(4):535-540.	Oncotype	15	≤ 40 years	1 (7%)	Retrospective – single center (4)
Wilson PC, Chagpar AB, Cicek AF, et al. Breast cancer histopathology is predictive of low-risk Oncotype Dx recurrence score. Breast J. 2018;24(6):976-980.	Oncotype†	371	≤ 40 years	4 (1%)	Retrospective – single center (4)
Siegelmann-Danieli N, Silverman B, Zick A, Beit-Or A, Katzir I, Porath A. The impact of the Oncotype DX Recurrence Score on treatment decisions and clinical outcomes in patients with early breast cancer: the Maccabi Healthcare Services experience with a	Oncotype*	751	18-44 years	138 (18%)	Prospective (2)

unified testing policy. Ecancermedicalscience. 2013;7:380.					
Albanell J, Gonzalez A, Ruiz-Borrego M, et al. Prospective transGEICAM study of the impact of the 21-gene Recurrence Score assay and traditional clinicopathological factors on adjuvant clinical decision making in women with estrogen receptor-positive (ER+) node-negative breast cancer. Ann Oncol. 2012;23(3):625-631.	Oncotype*	107	< 50 years	40 (37%)	Prospective – multicenter (1)
Frazier TG, Fox KR, Smith JS, et al. A retrospective study of the impact of 21-gene recurrence score assay on treatment choice in node positive micrometastatic breast cancer. Pharmaceuticals (Basel). 2015;8(1):107-122.	Oncotype*	181	< 50 years	26 (14%)	Retrospective – multicenter (2)
Solin LJ, Gray R, Goldstein LJ, et al. Prognostic value of biologic subtype and the 21-gene recurrence score relative to local recurrence after breast conservation treatment with radiation for early stage breast carcinoma: results from the Eastern Cooperative Oncology Group E2197 study. Breast Cancer Res Treat. 2012;134(2):683-692.	Oncotype*	388	< 50 years	170 (44%)	Prospective – multicenter (1)
Toi M, Iwata H, Yamanaka T, et al. Clinical significance of the 21-gene signature (Oncotype DX) in hormone receptor-positive early stage primary breast cancer in the Japanese population. Cancer. 2010;116(13):3112-3118.	Oncotype*	200	< 50 years	68 (34%)	Retrospective – multicenter (2)
Troester MA, Sun X, Allott EH, et al. Racial Differences in PAM50 Subtypes in the Carolina Breast Cancer Study. J Natl Cancer Inst. 2018;110(2).	Oncotype*	191	< 50 years	78 (41%)	Retrospective – registry-based (4)
Williams AD, Reyes SA, Arlow RL, Tchou J, De La Cruz LM. Is Age Trumping Genetic Profiling in Clinical Practice? Relationship of Chemotherapy Recommendation and Oncotype DX Recurrence Score in Patients Aged < 50 Years versus ≥ 50 Years, and Trends Over Time. Ann Surg Oncol. 2018;25(10):2875-2883.	Oncotype*	115,052	< 50 years	24,500 (21%)	Retrospective – registry-based (2)

Wolmark N, Mamounas EP, Baehner FL, et al. Prognostic Impact of the Combination of Recurrence Score and Quantitative Estrogen Receptor Expression (ESR1) on Predicting Late Distant Recurrence Risk in Estrogen Receptor-Positive Breast Cancer After 5 Years of Tamoxifen: Results From NRG Oncology/National Surgical Adjuvant Breast and Bowel Project B-28 and B-14. J Clin Oncol. 2016;34(20):2350-2358.	Oncotype*	565	< 50 years	221 (39%)	Prospective – multicenter (2)
Troester M, Sun X, Allott E, et al. Abstract PD8-01: Race and age differences in PAM50 biomarker status in the Carolina breast cancer study. Cancer Research. 2017;77(4 Supplement):PD8-01-PD08-01.	Oncotype*	191	≤ 50 years	78 (41%)	Retrospective – registry-based (4)
Nguyen MT, Stessin A, Nagar H, et al. Impact of oncotype DX recurrence score in the management of breast cancer cases. Clin Breast Cancer. 2014;14(3):182-190.	Oncotype*	407	≤ 50 years	126 (31%)	Retrospective – single center (2)
Penault-Llorca F, Filleron T, Asselain B, et al. The 21-gene Recurrence Score(R) assay predicts distant recurrence in lymph node-positive, hormone receptor-positive, breast cancer patients treated with adjuvant sequential epirubicin- and docetaxel-based or epirubicin-based chemotherapy (PACS-01 trial). BMC Cancer. 2018;18(1):526.	Oncotype*	530	≤ 50 years	250 (47%)	Prospective – multicenter (2)
Schneider JG, Khalil DN. Why does Oncotype DX recurrence score reduce adjuvant chemotherapy use? Breast Cancer Res Treat. 2012;134(3):1125-1132.	Oncotype*	89	≤ 50 years	31 (35%)	Retrospective – single center (2)
Cotter MB, Dakin A, Maguire A, et al. Comparison of Oncotype DX(R) Recurrence Score(R) with other risk assessment tools including the Nottingham Prognostic Index in the identification of patients with low-risk invasive breast cancer. Virchows Arch. 2017;471(3):321-328.	Oncotype*	300	≤ 50 years	95 (32%)	Retrospective – multicenter (4)
Sparano JA, Gray RJ, Ravdin PM, et al. Clinical and Genomic Risk to Guide the Use of Adjuvant Therapy for Breast Cancer. N Engl J Med. 2019;380(25):2395-2405.	Oncotype[†]	9,427	≤50 years	2,958 (31%)	Prospective – multicenter (1)
Sparano JA, Gray RJ, Makower DF, et al. Adjuvant Chemotherapy Guided by a 21-Gene Expression Assay in Breast Cancer. N Engl J Med. 2018;379(2):111-121.	Oncotype[†]	9,719	≤ 50 years	3,054 (31%)	Prospective – multicenter (1)

Yu-Qing Y, Lei W, Mei-Ling H, et al. Clinical significance of 21-gene recurrence score assay for hormone receptor-positive, lymph node-negative breast cancer in early stage. Exp Mol Pathol. 2019;108:150-155.	Oncotype*	222	≤ 50 years	135 (61%)	Retrospective – single center (2)
Holt S, Bertelli G, Humphreys I, et al. A decision impact, decision conflict and economic assessment of routine Oncotype DX testing of 146 women with node-negative or pNImi, ER-positive breast cancer in the U.K. Br J Cancer. 2013;108(11):2250-2258.	Oncotype*	142	< 55 years	67 (47%)	Prospective – single center (1)
Wu J, Fang Y, Lin L, et al. Distribution patterns of 21-gene recurrence score in 980 Chinese estrogen receptor-positive, HER2-negative early breast cancer patients. Oncotarget. 2017;8(24):38706-38716.	Oncotype*	896	≤ 55 years	442 (49%)	Retrospective – single center (2)
Gerson Cwilich R, Alban de la Torre LF, Villalobos Prieto A, Serrano Olvera JA. [Clinicopathological features, prognosis and influence in the adjuvant treatment of the risk recurrence groups determined by the 21 gene expression profile, Oncotype Dx(R), in early breast cancer]. Gac Med Mex. 2012;148(2):117-124.	Oncotype*	36	Premenopausal	17 (47%)	Prospective – single center (4)
Joh JE, Esposito NN, Kiluk JV, et al. The effect of Oncotype DX recurrence score on treatment recommendations for patients with estrogen receptor-positive early stage breast cancer and correlation with estimation of recurrence risk by breast cancer specialists. Oncologist. 2011;16(11):1520-1526.	Oncotype*	154	Premenopausal	65 (42%)	Retrospective – single center (2)
Khoury T, Huang X, Chen X, Wang D, Liu S, Opyrchal M. Comprehensive Histologic Scoring to Maximize the Predictability of Pathology-generated Equation of Breast Cancer Oncotype DX Recurrence Score. Appl Immunohistochem Mol Morphol. 2016;24(10):703-711.	Oncotype*	403	Premenopausal	130 (32%)	Retrospective-prospective – single center (4)
Muniz J, Kidwell KM, Henry NL. Associations between metabolic syndrome, breast cancer recurrence, and the 21-gene recurrence score assay. Breast Cancer Res Treat. 2016;157(3):597-603.	Oncotype*	528	Premenopausal	204 (39%)	Retrospective – single center (2)
Kelly CM, Krishnamurthy S, Bianchini G, et al. Utility of oncotype DX risk estimates in clinically intermediate risk hormone receptor-positive, HER2-normal, grade II, lymph node-negative breast cancers. Cancer. 2010;116(22):5161-5167.	Oncotype*	309	Premenopausal	101 (33%)	Prospective – single center (2)

Carr DN, Vera N, Sun W, et al. Menopausal status does not predict Oncotype DX recurrence score. J Surg Res. 2015;198(1):27-33.	Oncotype*	575	Premenoupausal	142 (25%)	Retrospective – single study (2)
Cardoso F, van't Veer LJ, Bogaerts J, et al. 70-Gene Signature as an Aid to Treatment Decisions in Early-Stage Breast Cancer. New England Journal of Medicine. 2016;375(8):717-729.	Mammaprint	6,693	< 35 years	122 (2%)	Prospective – multicenter (1)
Gevensleben H, Gohring UJ, Buttner R, et al. Comparison of MammaPrint and TargetPrint results with clinical parameters in German patients with early stage breast cancer. Int J Mol Med. 2010;26(6):837-843.	Mammaprint	140	≤ 35 years	2 (1%)	Retrospective (4)
Grant KA, Apffelstaedt JP, Wright CA, et al. MammaPrint Pre-screen Algorithm (MPA) reduces chemotherapy in patients with early-stage breast cancer. S Afr Med J. 2013;103(8):522-526.	Mammaprint	104	≤ 35 years	4 (4%)	Cross-sectional (1)
Bueno-de-Mesquita JM, van Harten WH, Retel VP, et al. Use of 70-gene signature to predict prognosis of patients with node-negative breast cancer: a prospective community-based feasibility study (RASTER). Lancet Oncol. 2007;8(12):1079-1087.	Mammaprint	427	< 40 years	67 (16%)	Prospective – multicenter (1)
Groenendijk FH, Jager A, Cardoso F, van Deurzen CHM. A nationwide registry-based cohort study of the MammaPrint genomic risk classifier in invasive breast cancer. Breast. 2018;38:125-131.	Mammaprint	1,946	< 40 years	59 (3%)	Retrospective – registry-based (4)
Mook S, Schmidt MK, Viale G, et al. The 70-gene prognosis-signature predicts disease outcome in breast cancer patients with 1-3 positive lymph nodes in an independent validation study. Breast Cancer Res Treat. 2009;116(2):295-302.	Mammaprint	241	< 40 years	23 (10%)	Retrospective – multicenter (2)
Wittner BS, Sgroi DC, Ryan PD, et al. Analysis of the MammaPrint breast cancer assay in a predominantly postmenopausal cohort. Clin Cancer Res. 2008;14(10):2988-2993.	Mammaprint	100	< 40 years	4 (4%)	Retrospective – single center (2)
Nunes RA, Wray L, Mete M, et al. Genomic profiling of breast cancer in African-American women using MammaPrint. Breast Cancer Res Treat. 2016;159(3):481-488.	Mammaprint	100	≤ 40 years	9 (9%)	Prospective-retrospective – single center (4)
Buyse M, Loi S, van't Veer L, et al. Validation and clinical utility of a 70-gene prognostic signature for	Mammaprint	302	≤ 40 years	58 (19%)	Retrospective – multicenter (2)

women with node-negative breast cancer. J Natl Cancer Inst. 2006;98(17):1183-1192.					
Aalders K, Genbrugge E, Poncet C, et al. Abstract P1-07-08: Young age and the risk of disease recurrence as assessed by the 70-gene signature – an analysis from the EORTC 10041/BIG 03-04 MINDACT trial. Cancer Research. 2018;78(4 Supplement):P1-07-08-P01-07-08.	Mammaprint	6,693	< 45 years	1,100 (2%)	Prospective – multicenter (2)
Dabbs DJ, Brufsky A, Jankowitz RC, et al. Comparison of test results and clinical outcomes of patients assessed with both MammaPrint and Oncotype DX with pathologic variables: An independent study. Journal of Clinical Oncology. 2014;32(15_suppl):550-550.	Mammaprint	437	< 50 years	105 (24%)	Retrospective – single center (2)
Ishitobi M, Goranova TE, Komoike Y, et al. Clinical Utility of the 70-gene MammaPrint Profile in a Japanese Population. Japanese Journal of Clinical Oncology. 2010;40(6):508-512.	Mammaprint	102	< 50 years	34 (33%)	Retrospective – single study (2)
Saghatchian M, Mook S, Pruneri G, et al. Additional prognostic value of the 70-gene signature (MammaPrint((R))) among breast cancer patients with 4-9 positive lymph nodes. Breast. 2013;22(5):682-690.	Mammaprint	173	≤ 50 years	95 (55%)	Retrospective – multicenter (2)
Soliman H, Lo S, Qamar R, et al. Abstract P4-08-10: MammaPrint identifies 46% of patients, age ≤50 years with oncotype RS 18-30, as low risk and safe to forgo chemotherapy. Cancer Research. 2019;79(4 Supplement):P4-08-10-P04-08-10.	Mammaprint	840	≤ 50 years	181 (22%)	Prospective – multicenter (4)
Straver ME, Glas AM, Hannemann J, et al. The 70-gene signature as a response predictor for neoadjuvant chemotherapy in breast cancer. Breast Cancer Res Treat. 2010;119(3):551-558.	Mammaprint	167	Premenopausal	119 (71%)	Observational – single center (2)
Walter VP, Taran FA, Wallwiener M, et al. A high-risk 70-gene signature is not associated with the detection of tumor cell dissemination to the bone marrow. Breast Cancer Res Treat. 2018;169(2):305-309.	Mammaprint	149	Premenopausal	50 (34%)	Retrospective – single center (4)
Mokbel K, Wazir U, Wazir A, Kasem A. The Impact of EndoPredict Clinical Score on Chemotherapy Recommendations in Women with Invasive ER(+)/HER2(-) Breast Cancer Stratified as Having Moderate or Poor Prognosis by Nottingham	EndoPredict	120	≤ 40 years	4 (3%)	Prospective – single center (4)

Prognostic Index. Anticancer Res. 2018;38(8):4747-4752.					
Villarreal-Garza C, Deneken-Hernandez Z, Maffuz-Aziz A, et al. Abstract P2-08-54: Change in therapeutic management after EndoPredict assay in a prospective decision impact study of Mexican premenopausal patients. Cancer Research. 2019;79(4 Supplement):P2-08-54-P02-08-54.	EndoPredict	91	≤ 40 years	30 (33%)	Prospective – multicenter (2)
Bertucci F, Finetti P, Viens P, Birnbaum D. EndoPredict predicts for the response to neoadjuvant chemotherapy in ER-positive, HER2-negative breast cancer. Cancer Lett. 2014;355(1):70-75.	EndoPredict	553	≤ 50 years	298 (54%)	Retrospective – multicenter (2)
Pelaez-Garcia A, Yebenes L, Berjon A, et al. Comparison of risk classification between EndoPredict and MammaPrint in ER-positive/HER2-negative primary invasive breast cancer. PLoS One. 2017;12(9):e0183452.	EndoPredict	40	≤ 55 years	17 (42%)	Retrospective – single center (4)
Martin M, Brase JC, Calvo L, et al. Clinical validation of the EndoPredict test in node-positive, chemotherapy-treated ER+/HER2− breast cancer patients: results from the GEICAM 9906 trial. Breast Cancer Research. 2014;16(2):R38.	EndoPredict	555	Premenopausal	300 (54%)	Prospective – multicenter (1)
Liedtke C, Hatzis C, Symmans WF, et al. Genomic grade index is associated with response to chemotherapy in patients with breast cancer. J Clin Oncol. 2009;27(19):3185-3191.	GGI	229	≤ 50 years	118 (52%)	Prospective – multicenter (2)
Naoi Y, Kishi K, Tanei T, et al. High genomic grade index associated with poor prognosis for lymph node-negative and estrogen receptor-positive breast cancers and with good response to chemotherapy. Cancer. 2011;117(3):472-479.	GGI	189	Premenopausal	90 (48%)	Retrospective-prospective (2)
Mathieu MC, Mazouni C, Kesty NC, et al. Breast Cancer Index predicts pathological complete response and eligibility for breast conserving surgery in breast cancer patients treated with neoadjuvant chemotherapy. Ann Oncol. 2012;23(8):2046-2052.	BCI	150	≤ 50 years	66 (44%)	Retrospective – single center (2)

*The Oncotype DX recurrence score thresholds used for defining low, intermediate and high risk of recurrence were <18, 18-30 and >30.

†The Oncotype DX recurrence score thresholds used for defining low, intermediate and high risk of recurrence were <11, 11-25 and >25.
